# Exchange Interactions on the Highest-Spin Reported Molecule: the Mixed-Valence Fe_42_ Complex

**DOI:** 10.1038/srep23847

**Published:** 2016-04-01

**Authors:** Daniel Aravena, Diego Venegas-Yazigi, Eliseo Ruiz

**Affiliations:** 1 Universidad de Santiago de Chile (USACH), Departamento de Química de los Materiales, Facultad de Química y Biología, Santiago de Chile, Chile; 2 CEDENNA, Santiago, Chile; 3 Universitat de Barcelona, Departament de Química Inorgànicai Orgànica and Institut de Recerca en Química Teòrica i Computacional, Barcelona, 08028, Spain

## Abstract

The finding of high-spin molecules that could behave as conventional magnets has been one of the main challenges in Molecular Magnetism. Here, the exchange interactions, present in the highest-spin molecule published in the literature, Fe_42_, have been analysed using theoretical methods based on Density Functional Theory. The system with a total spin value S = 45 is formed by 42 iron centres containing 18 high-spin Fe^III^ ferromagnetically coupled and 24 diamagnetic low-spin Fe^II^ ions. The bridging ligands between the two paramagnetic centres are two cyanide ligands coordinated to the diamagnetic Fe^II^ cations. Calculations were performed using either small Fe_4_ or Fe_3_ models or the whole Fe_42_ complex, showing the presence of two different ferromagnetic couplings between the paramagnetic Fe^III^ centres. Finally, Quantum Monte Carlo simulations for the whole system were carried out in order to compare the experimental and simulated magnetic susceptibility curves from the calculated exchange coupling constants with the experimental one. This comparison allows for the evaluation of the accuracy of different exchange-correlation functionals to reproduce such magnetic properties.

A major goal in the field of Molecular Magnetism is the synthesis of molecules that can play a similar role to conventional magnet (metals or alloys). These molecular systems can provide with new functionalities, such as solubility, photochemical properties and lighter storage units among others^1^,^2^. The discovery in 1992 of the single-molecule magnet (SMM) behaviour of the Mn_12_ molecule, which behaves as a magnet at very low temperatures, directed the search towards high-spin molecules^3^,^4^. The energy barrier that fixes the orientation of the spin providing the behaviour as a magnet is equal to |D|·S^2^; being D the the zero-field splitting parameter and S the total spin value^5^. Over the years, the quest to increase the blocking temperature of only 2 K for the Mn_12_ (S = 10)^6^ has lead towards the syntheses of a large variety of polynuclear complexes with ferromagnetic or ferrimagnetic interactions, aiming to achieve larger values of the total spin^7^,^8^. Thus, reaching high-spin molecules has been one of the main challenges in this research field. Few years after the discovery of the SMM behaviour, in 1995, a Fe_19_ complex was characterized with S = 33/2 being the spin record during the following five years^9^,^10^. This value was surpassed in 2000 by some heteronuclear Mn_9_M_6_ (M = Mo and W) complexes with a total spin of 39/2, respectively^11^,^12^. Initially, a spin of 51/2 was assigned to one of them, the Mn_9_W_6_ system, although lately it was corroborated the ferromagnetic nature of the interactions by using theoretical methods with a resulting value of 39/2 for the total spin as well as its analogous with Mo^13^. Few years later, in 2004, the value of S = 51/2 was reached by a Mn_25_ complex containing one Mn^IV^, eighteen Mn^III^ and six Mn^II^ centres^14^. It is worth noting that in such system, the maximum expected S value for a parallel alignment of all the spins would give an S = 105/2. Thus, ferrimagnetic or antiferromagnetic interactions were assumed within the complex. Lately in 2007, the same research group reported another Mn_25_ complex with a total spin of 61/2 by replacing the azido ligands of the aforementioned Mn_25_ complex by N,O-chelating groups^15^. However, all the above S values were significantly surpassed in 2006 by a Mn_19_ complex, reaching the maximum spin value of 83/2, consistent with a ferromagnetic coupling between twelve Mn^III^ and seven Mn^II^ centres^16^,^17^. This Mn_19_ system prevailed during years as the highest-spin reported molecule until the publication in 2015 by Kang and coworkers of an Fe_42_ complex with twenty-four diamagnetic Fe^II^ cations and eighteen Fe^III^ centres ferromagnetically coupled, resulting in a S value of 45 (90/2)^18^. It is worth to mention that all the complexes exposed so far, despite the large S values, did not present exceptional SMM properties, cancelled out by the presence of small magnetic anisotropy values in all of them^19^,^20^. For instance, the Mn_19_ complex did not exhibit SMM behaviour^21^ and the SMM with the highest reported spin, since 2009, is a ferromagnetically-coupled manganese complex (Mn_17_) with eleven Mn^III^ and six Mn^II^ centres resulting in an overall S = 37 value^22^,^23^. Concerning the Fe_42_, magnetic characterization of the recently-synthesized Fe_42_ complex did not include AC measurements to determine the SMM behaviour^18^, probably unexpected due to the isotropic nature of the Fe^III^ centres.

The Fe_42_ complex, [{Fe(Tp)(CN)_3_}_24_{Fe(H_2_O)_2_}_6_{Fe(dpp)(H_2_O)}_12_·6CF_3_SO_3_]·18H_2_O (where dpp = 1,3-di(4-pyridyl)propane, and Tp = hydrotris(pyrazolyl)borate), with the reported value of S = 45 has a singular structure (see Fig. 1)^18^, where the cyanide bridging ligands have Fe^II^-CN-Fe^III^ coordination and all the Fe^II^ cations coordinated to the bridging ligand through the C atom, meanwhile the nitrogen atom is always attached to the Fe^III^ centres. Thus, such coordination mode does not follow hard-soft criterion. The usual coordination, Fe^III^-CN-Fe^II^, was also obtained by the same authors in a previous system that exhibited single-chain magnetic behaviour and light-induced spin crossover properties, due to the coordination of the Fe^II^ cations with a total of six nitrogen atoms^24^. Back to Fe_42_, the coordination of the Fe^III^ cations with the nitrogen atoms, instead of carbon atoms, is key in reaching the local S = 5/2 high-spin for each Fe^III^ centre, thus allowing the high-spin state for the molecule, equivalent to that found in the well-known Prussian blue structures Fe^III^_4_[Fe^II^(CN)_6_]_3_·xH_2_O^25^.

Some theoretical studies were performed within the original paper^18^, but our main goal here is to carry out a complete study of the exchange interactions in the Fe_42_ complex, which has not been performed up to date. Both small models and the whole molecule have been employed to analyse the exchange interactions using DFT calculations. The big challenges here are to proceed with the calculations for the whole molecule, with a total of 1230 atoms (represented in Fig. 1), together with the difficulties of performing open-shell calculations with several paramagnetic centres. Furthermore, we considered all the exchange interactions present in the system together with the use of Quantum Monte Carlo (QMC) simulations^26^ (the large number of paramagnetic centres present in the system avoid the use of exact diagonalization approach) to compare the magnetic susceptibility extracted from theoretical methods with the experimental one. Our findings indicated that the two-types of exchange interactions present in the system are ferromagnetic and such values theoretically corroborate the S = 45 total spin experimentally reported. This agreement is particularly relevant because of the difficult task of correctly asses magnetic characterizations of high spin molecules with theoretical methods have been a crucial tool to support experimental results.

## Results

**Figure 1:**
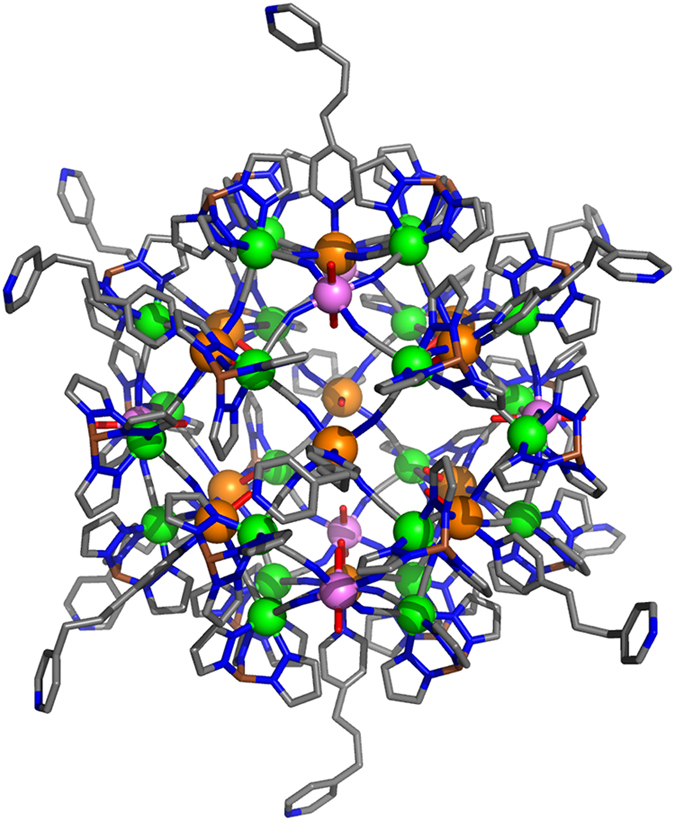
Structure of the Fe_42_ complex [{Fe(Tp)(CN)_3_}_24_{Fe(H_2_O)_2_}_6_{Fe(*dpp*)(H_2_O)}_12_]^6+^ employed in the calculations (see Methods section). There are two types of S = 5/2 Fe^III^ cations represented by orange and violet spheres, respectively while the diamagnetic Fe^II^ centres are indicated as green spheres. Boron, carbon, nitrogen and oxygen atoms are represented by brow, gray, blue and red cylinders and hydrogen atoms are omitted for clarity.

**Figure 2:**
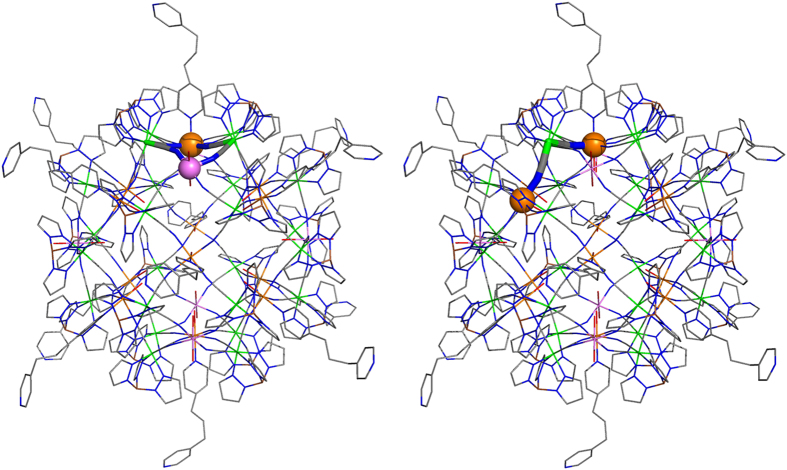
Description of the two exchange interaction pathways between the Fe^III^ cations in the Fe_42_ complex (left, J_1_ and right J_2_). Only the atoms involved in the pathway are plotted with the ball-cylinder representation while the rest of the molecule is represented as a wireframe. Fe^III^ cations represented by orange and violet colours and diamagnetic Fe^II^ centres as green. Boron, carbon, nitrogen and oxygen atoms are represented by brown, gray, blue and red colours and hydrogen atoms are omitted for clarity.

The magneto-structural analysis of the Fe_42_ reveals the presence of two different first-neighbour exchange interaction pathways between the Fe^III^ cations (see Fig. 2). Hence, there are two types of Fe^III^ centres: one Fe^III^ cation is equatorially coordinated by four nitrogen atoms of cyanide groups, one H_2_O molecule and one *dpp* ligand both in axial positions (type 1, orange spheres in Fig. 1); and a second class of Fe^III^ centres similar to type 1 but containing two water molecules in the axial positions (type 2, violet spheres in Fig. 1). The first exchange interaction J_1_ (left in Fig. 2) corresponds to the interaction between the two-types of Fe^III^ cations (Fe^III^···Fe^III^ distance of 6.74 Å) through a double NC-Fe^II^-CN bridging ligand (Fe^III^···Fe^II^···Fe^III^ angles of 85.7 and 86.1°, respectively). The second exchange pathway, J_2_, was defined between the two type 1 Fe^III^ centres (Fe^III^···Fe^III^ distance of 7.84 Å) (Fig. 2, right) and is mediated by a single NC-Fe^II^-CN bridging ligand (Fe^III^···Fe^II^···Fe^III^ angle of 102.9°). The second-neighbour interactions between Fe^III^ cations have distances longer than 11 Å and therefore was not considered here. In summary, twelve type 1 Fe^III^ cations surrounded each one by two type 2 Fe^III^ cations results in a total of 24 J_1_ interactions and 24 J_2_ interactions for the whole Fe_42_ complex (see Supplementary information for the detailed spin Hamiltonian). Thus, a total of 48 exchange interactions were used in the Fe_42_ complex but due to the cubic symmetry of the crystal structure (space group Pn-3n) many interactions are equivalent, thus leading to only two different exchange coupling constants.

DFT calculations were performed using the FHI-aims code (see details in Methods section) for the whole structure represented in Fig. 1 and for two models, Fe_4_ and Fe_3_, corresponding to the two exchange pathways highlighted in Fig. 2 adding the terminal ligands of the iron centres. The calculated J values are collected in Table 1. From these results, we can extract the following conclusions: (i) all the calculated coupling constants are ferromagnetic, thus, they will provide a S = 45 ground state. (ii) The PBE functional provides with relatively stronger ferromagnetic interactions than the hybrid functionals. Clearly, the increase in the Hartree-Fock type exchange contribution results in a decrease of the calculated ferromagnetic J values (0% contribution for PBE, 20% for B3LYP and 50% for the HSE06). (iii) The exchange interaction J_1_ through the double NC-Fe^II^-CN bridging ligands seems to be more ferromagnetic than J_2_, which is described as a single bridge, when the full Fe_42_ structure is used in the calculations. (iv) In the reduced models, the presence of only one or two single bridging ligand causes a substantial spin delocalization towards such bridging ligand (NC-Fe^II^-CN), resulting in an overestimation of the exchange coupling constants with the PBE functional (see Table 1). Hence, the Fe_3_ and Fe_4_ reduced models must be employed with caution because they can provide significant differences in the calculated J values compared to those obtained with the whole Fe_42_ system.

**Figure 3:**
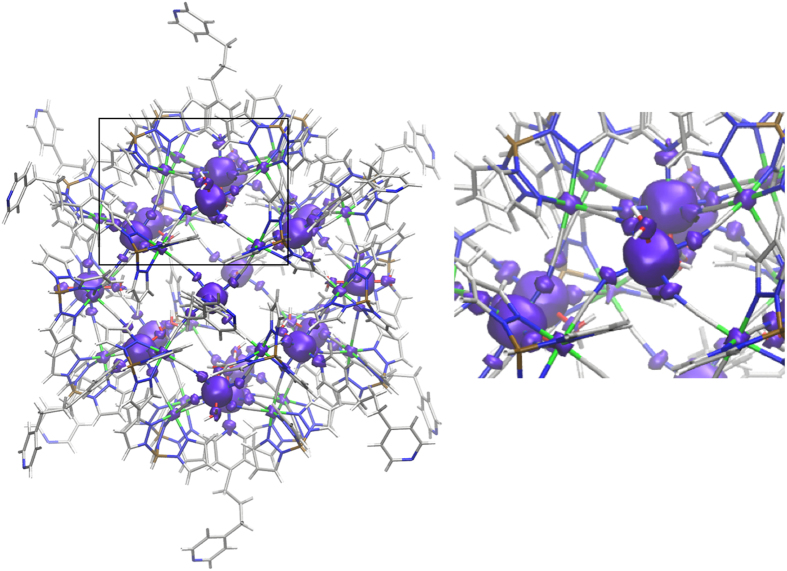
(left) Representation of the spin density for the S = 45 ground state of the Fe_42_ complex calculated with the HSE06 functional and (right) inset of the region enclosed in the rectangle of the left figure. Violet (positive) isosurface of 0.05 e^−^/bohr^3^. Negative values are below such threshold. Fe^II^ centres are represented as green while boron, carbon, nitrogen and oxygen atoms are represented by brown, gray, blue and red colours, respectively.

The spin density of the S = 45 ground state for the whole Fe_42_ complex calculated with the HSE06 functional is represented in Fig. 3. The Fe^III^ cations have almost spherical densities due to the high-spin t_2g_^3^e_g_^2^ orbital occupation. The presence of two unpaired electrons in the antibonding e_g_ orbitals produces a predominant delocalization mechanism of the spin density (on the coordinated nitrogen atoms, see inset in Fig. 3) over the spin polarization one, being the latest induced by the t_2g_ orbitals^27^,^28^. Hence, first coordination sphere atoms have their spin densities with the same sign than the metallic centre. It is worth noting that the relatively small spin population on the Fe^II^ centres (see inset in Fig. 3, around 0.03 e− with the HSE06 functional) is close to the proposed value for the analogous Prussian blue structure obtained using polarized neutron diffraction^29^. The significant decrease in the spin population found on the Fe^III^ cations (around 4.4 e^−^) in comparison with the formal expected value of five unpaired electrons is due to the spin delocalization within the ligands. The spin population values can also be employed to quantify the above mentioned problem about the use of structural models. It is well-known that GGA functionals, for instance PBE, usually overestimate the spin delocalisation^30^. The truncation of the full structure to obtain a small model induces an unrealistic large spin densities on the few (one or two) bridging Fe^II^ centres considered in such models (mainly with the PBE functional, see Fig. S2 showing a linear correlation between all DFT calculated J constants for the Fe_3_ and Fe_4_ models and the Fe^II^ spin population values). Only the HSE06 functional results in a similar Fe^II^ spin population values independently of the structural model. In addition, for mixed-valence systems, hybrid B3LYP functional produces a high electron (and spin) transfer on the diamagnetic Fe^II^ centres. Hence, these two factors, small structural models and the choice of functional, yields to an overestimation of the calculated J values for the models, being the worst studied case the one involving the PBE functional and Fe_3_ model, due to a large spin delocalization on the Fe^II^ centres (see Fig. S2).

## Discussion

**Figure 4:**
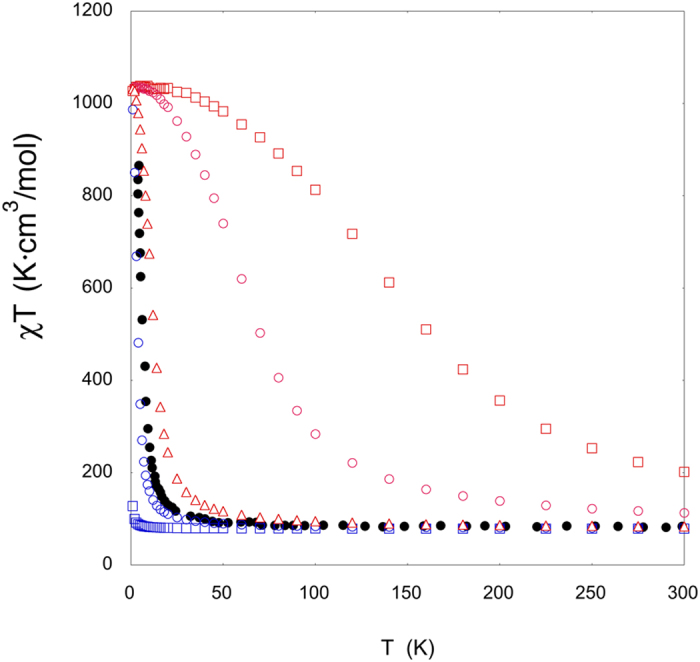
Temperature dependence of χT product. Experimental data is represented with black circles, while those obtained with QMC simulations using the calculated DFT J values for the whole structure are indicated with red symbols (squares – PBE, circles – B3LYP and triangles – HSE06). The values with blue symbols are the QMC simulations with a single J value extracted with the mean-field expressions (circles J_1_ = +0.57 cm^−1^ using Langevin, Weiss and Néel equation, squares J_1_ = +0.04 cm^−1^ value fitted by Kang and coworkers) .

In this section, the main goal is to determine the accuracy of the calculated J values by comparison with the experimental data. Thus, we performed Quantum Monte Carlo simulations (see details in Method section) using the DFT J values, aiming to achieve a magnetic susceptibility curve that can be directly compared with the experimental curve (see Fig. 4). The comparison shows a large overestimation for the calculated ferromagnetic coupling when the PBE and the hybrid B3LYP functionals are used with whole system; however, the screened hybrid HSE06 functional is in excellent agreement with the experimental data. Despite that there are many examples in the literature showing that B3LYP functional gives excellent results for the calculation of exchange coupling constants in non mixed-valence systems^31^,^32^,^33^,^34^. The failure of the B3LYP functional in our study is due to the extremely large spin delocalization on the Fe^II^ centres, which causes an unrealistic electronic structure, resulting in an overestimation of the calculated J values. Hence, this drawback of some functionals to describe the electron (or spin) delocalization should be especially important in mixed-valence systems despite that they can provide accurate J values, as B3LYP functional, in non mixed-valence complexes. This fact was also previously noticed in some mixed-valence systems showing a too small electron-transfer matrix elements calculated with the B3LYP functional^35^.

It is also worth mentioning that the theoretical analysis using B3LYP* functional^36^ and the Fe_4_ model calculations (neglecting the J_2_ interaction) performed in the original paper^18^ resulted in a very large ferromagnetic J_1_ value (+35.5 cm^−1^ with the spin projected approach). The authors considered the energy difference between the high-spin S = 5 state for the Fe_4_ model with a “broken-symmetry” S = 0 solution but with a low-spin S = 1/2 for each of the two Fe^III^ centres, instead of just the inversion of one 5/2 local spin.

Despite the improvement obtained using the screened HSE06 hybrid functional, the ferromagnetic exchange constants were slightly larger than the experimental data. As an alternative to estimate the J value from the experimental data, we used the approximate mean-field expression derived from Langevin, Weiss and Néel^37^,^38^:


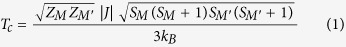


which provides a bridge between the experimentally determined Curie Temperature T_C_ (6.6 K for the Fe_42_ complex, similar to that of the Prussian blue Fe^III^_4_[Fe^II^(CN)_6_]_3_·xH_2_O system^25^ of 6 K) and the computable exchange coupling constant between nearest M and M’ neighbours, J. Here, S_M_ and S_M’_ are the local spins (S = 5/2 for Fe^III^ cations), and Z_M_ and Z_M’_ the number of nearest neighbours of each type of metal atom (2 and 4, respectively). This approach neglects the J_2_ interaction, thus providing a J_1_ value of +0.57 cm^−1^. The QMC simulations performed with such J_1_ value are in very good agreement with the experimental data. It is worth noting that the mean field approach employs the experimental T_C_ value to calculate J_1_ value while DFT methods are an *ab initio* strategy, there are neither experimental parameters nor scaling factors. Kang and coworkers also performed a fit of the experimental data using a very crude estimation of the state energies for the Fe_42_ system as function of the J value^18^. This procedure provides with a very small J value of +0.04 cm^−1^ that logically results in a magnetic susceptibility curve that is far away from the experimental results (Fig. 4).

## Methods

DFT calculations were performed with the all-electron FHI-aims computer code using numerical local orbital basis set^39^. This approach allows for a full-potential calculations at a low computational cost without using any a priori approximations for the potential, such as pseudopotentials or frozen cores. The calculations of the whole Fe_42_ complex and Fe_3_ and Fe_4_ models were performed using the generalized-gradient approximation PBE functional^40^ as well as the hybrid B3LYP^41^ and screened hybrid HSE06 functionals^42^,^43^. For the HSE06 functional, we have selected the positive screening parameter ω = 0.25 with mixing parameter (Hartree-Fock type exchange) of 0.5 for the short-range exchange^44^. In the FHI-aims code, there are three levels of accuracy in the choice of the basis set (“light”, “tight” and “really tight”). Due to the lack of reported studies of the exchange interaction using FHI-aims, we performed test calculations with the Fe_4_ model and PBE functional. The calculated J_1_ value of +13.7 cm^−1^ at “light” level changed only in +0.1 cm^−1^ when the “tight” and “really tight” basis sets were employed. Thus, the numerical “light” basis set was employed in the all calculations presented in the paper. The SCF parameters to reach a good convergence in the calculations were a Gaussian occupation type with a parameter of 0.01, a Pulay mixer with 15 cycles and a mixing parameter of 0.04.

The experimental geometry obtained a 100 K (there is a second structure at room temperature) was employed for all DFT calculations. Disordered atomic positions in the *ddp* ligand and hydrogen atoms of the water molecules were optimised using a molecular mechanics approach with the universal force field^45^. It is important to stress that force-field optimisations are not associated with any change to the metal centres and their coordination sphere from the experimental structure. The J values were calculated for the Fe_3_ and Fe_4_ models that contain two paramagnetic Fe^III^ centres as the energy difference between the high-spin S = 5 and the “broken-symmetry” S = 0 solution divided by a factor 15 (non-spin projected approach). In order to extract the two J values for the whole structure, we performed five calculations the high spin S = 45 state, one S = 15 solution with the spin inversion of the six {13–18} type 2 Fe^III^ centres and three S = 35 solutions with the spin inversion of {1, 4}, {13, 16} and {1, 6} centres (see atom labels and spin Hamiltonian in Supplementary information). A detailed description of the mathematical procedure to determine the exchange coupling constants for dinuclear and polynuclear metal complexes can be found in previous works^31^,^32^,^33^,^34^. The two J values were calculated by a least-square fitting of the four equations resulting of the energy differences between the five employed spin distributions.

The usual procedure to check the accuracy of the calculated J values is the generation of the χT curves for comparison with the experimental data. The best procedure for obtaining such curves is to perform exact diagonalisation of the Hamiltonian. However, this approach presents a quick scaling in terms of computational resources, with a practical limit of ten S = 5/2 paramagnetic centres in our infrastructure. Thus, it is necessary to use approximate methods in order to perform a comparison with the experimental data. Quantum Monte Carlo methods represent an excellent alternative. Quantum Monte Carlo simulations based on the directed loop algorithm method developed by Sandvik *et al.*^46^ were performed using the ALPS 2.0 library^26^. The initial 10% of steps were employed for thermalisation of the system in all calculations. A total of 10^8^ steps were employed in order to reach the convergence of the simulations using the theoretically calculated J values.

## Additional Information

**How to cite this article**: Aravena, D. *et al.* Exchange Interactions on the Highest-Spin Reported Molecule: the Mixed-Valence Fe_42_ Complex. *Sci. Rep.* **6**, 23847; doi: 10.1038/srep23847 (2016).

## Supplementary Material

Supplementary Information

## Figures and Tables

**Table 1:** Calculated exchange coupling constants in cm^−1^ for the Fe_42_ complex and the Fe_3_ and Fe_4_ model structures using PBE, B3LYP and HSE06 functionals with the corresponding bridging ligands and Fe···Fe distances in Å also indicated.

	_d (Fe···Fe)_	_Bridging ligand_	_Model_			_Full_		
			_J_PBE__	_J_B3LYP__	_J_HSE06__	_J_PBE__	_J_B3LYP__	_J_HSE06__
J_1_	_6.74_	double NC-Fe^II^-CN	+13.7	+2.8	+0.37	+13.1	+5.7	+0.90
J_2_	_7.84_	single NC-Fe^II^-CN	+20.6	+6.2	+0.33	+7.2	+3.1	+0.66

The two employed model systems (see Fig. 2) for the J_1_ interaction is a Fe_4_ model (Fe^III^_2_ Fe^II^_2_) while for the J_2_ case is a Fe_3_ model (Fe^III^_2_ Fe^II^) and the full structure corresponds to the cation represented in Fig. 1.
