# Supplementary material for: Exchange Interactions on the Highest-Spin Reported Molecule: the Mixed-Valence Fe42 Complex
Source: Sci Rep. 2016 Apr 1;6:23847. doi: 10.1038/srep23847 (PMC4817144; doi:10.1038/srep23847)

# Exchange Interactions on the Highest-Spin Reported Molecule: the Mixed-Valence $Fe_{42}$ Complex

**Daniel Aravena,[1+] Diego Venegas-Yazigi[1,2+] and Eliseo Ruiz[3 *]**

[1] Universidad de Santiago de Chile (USACH), Departamento de Química de los Materiales, Facultad de Química y Biología, Santiago de Chile, Chile

[2] CEDENNA, Santiago, Chile.

[3]Universitat de Barcelona, Departament de Química Inorgànica and Institut de Recerca en Química Teòrica i Computacional, Barcelona, 08028, Spain.

[*] eliseo.ruiz@qi.ub.es

## Supplementary Information

The spin Hamiltonian spin for a general polynuclear complex is indicated in Eq. S1,

$$\hat{H} = -\sum_{i>j} J_{ij}\ \hat{S}_i \hat{S}_j \qquad \text{(S1)}$$

where $\hat{S}_i$ and $\hat{S}_j$ are the spin operators of the paramagnetic centres *i* and *j*. In the case of the $Fe_{42}$ complex with the two exchange coupling constants $J_1$ and $J_2$ defined in Fig. 2, the spin Hamiltonian has the following expression (see Figure S1):

$$\begin{aligned} \hat{H} = -J_1\ (&\hat{S}_1\hat{S}_{13} + \hat{S}_1\hat{S}_{14} + \hat{S}_2\hat{S}_{13} + \hat{S}_2\hat{S}_{17} + \hat{S}_3\hat{S}_{14} + \hat{S}_3\hat{S}_{16} + \\ &\hat{S}_4\hat{S}_{16} + \hat{S}_4\hat{S}_{17} + \hat{S}_5\hat{S}_{14} + \hat{S}_5\hat{S}_{18} + \hat{S}_6\hat{S}_{13} + \hat{S}_6\hat{S}_{15} + \\ &\hat{S}_7\hat{S}_{13} + \hat{S}_7\hat{S}_{18} + \hat{S}_8\hat{S}_{14} + \hat{S}_8\hat{S}_{15} + \hat{S}_9\hat{S}_{15} + \hat{S}_9\hat{S}_{17} + \\ &\hat{S}_{10}\hat{S}_{17} + \hat{S}_{10}\hat{S}_{18} + \hat{S}_{11}\hat{S}_{15} + \hat{S}_{11}\hat{S}_{16} + \hat{S}_{12}\hat{S}_{16} + \hat{S}_{12}\hat{S}_{18}) \\ -J_2\ (&\hat{S}_1\hat{S}_5 + \hat{S}_1\hat{S}_6 + \hat{S}_1\hat{S}_7 + \hat{S}_1\hat{S}_8 + \hat{S}_2\hat{S}_6 + \hat{S}_2\hat{S}_7 + \\ &\hat{S}_2\hat{S}_9 + \hat{S}_2\hat{S}_{10} + \hat{S}_3\hat{S}_5 + \hat{S}_3\hat{S}_8 + \hat{S}_3\hat{S}_{11} + \hat{S}_3\hat{S}_{12} + \\ &\hat{S}_4\hat{S}_9 + \hat{S}_4\hat{S}_{10} + \hat{S}_4\hat{S}_{11} + \hat{S}_4\hat{S}_{12} + \hat{S}_5\hat{S}_7 + \hat{S}_5\hat{S}_{12} + \\ &\hat{S}_6\hat{S}_8 + \hat{S}_6\hat{S}_9 + \hat{S}_7\hat{S}_{10} + \hat{S}_8\hat{S}_{11} + \hat{S}_9\hat{S}_{11} + \hat{S}_{10}\hat{S}_{12}) \end{aligned} \qquad \text{(S2)}$$

**Figure S1.** Structure of the $Fe_{42}$ indicating the labels of the $Fe^{III}$ centres (orange and violet spheres) employed to define the spin Hamiltonian. $J_1$ interaction correspond to the exchange between type 1 $Fe^{III}$ (orange, label 1-12) and type 2 $Fe^{III}$ (violet, label 1-12) cations while $J_2$ constants involved interactions between type 1 $Fe^{III}$ (orange, label 1-12).

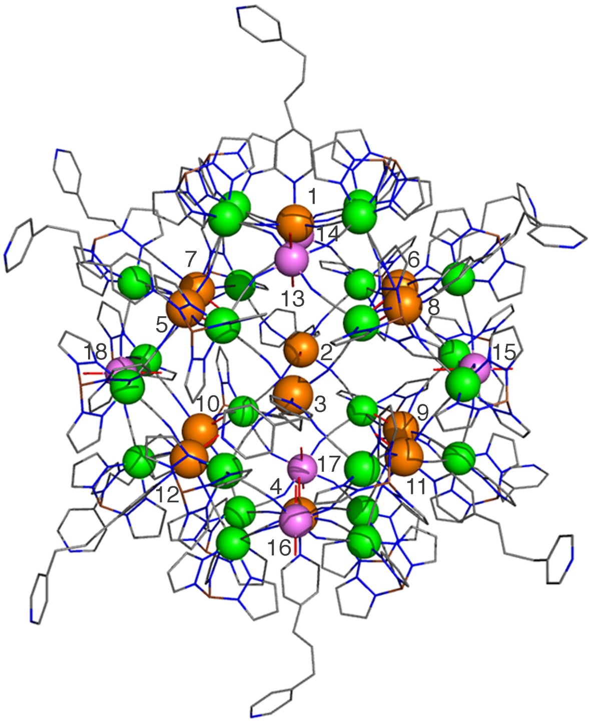

**Figure S2.** Dependence of the DFT calculated (PBE squares, B3LYP circles and HSE06 triangles) J values with the spin population (in electrons) of the bridging $Fe^{II}$ centres using $Fe_4$ and $Fe_3$ model structures.

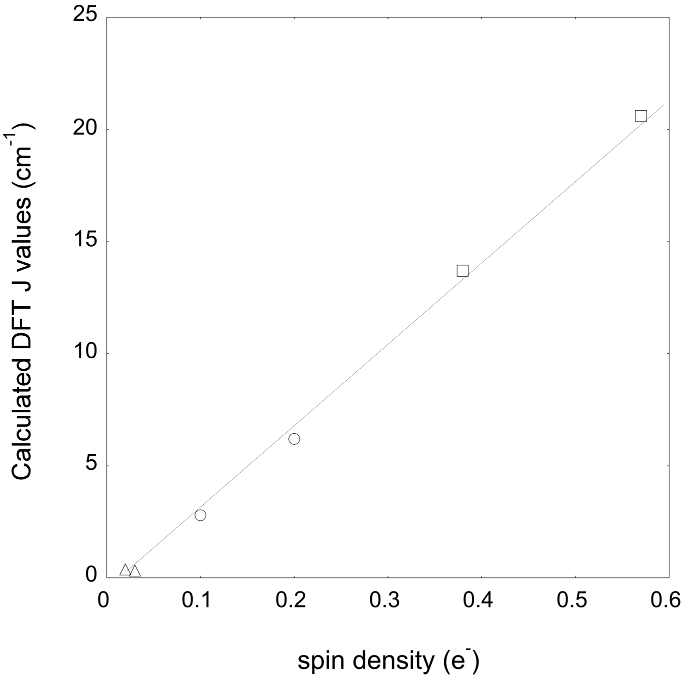

Supplement: Supplementary Information [file srep23847-s1.pdf]
